# Effects of Sport-Based Interventions on Executive Function in Older Adults: A Systematic Review and Meta-Analysis Protocol

**DOI:** 10.3390/brainsci12091142

**Published:** 2022-08-27

**Authors:** Falonn Contreras-Osorio, Rodrigo Ramirez-Campillo, Enrique Cerda-Vega, Rodrigo Campos-Jara, Cristian Martínez-Salazar, Cristián Arellano-Roco, Christian Campos-Jara

**Affiliations:** 1 Exercise and Rehabilitation Sciences Institute, Faculty of Rehabilitation Sciences, Universidad Andres Bello, Santiago 7591538, Chile; 2 Exercise and Rehabilitation Sciences Institute, School of Physical Therapy, Faculty of Rehabilitation Sciences, Universidad Andres Bello, Santiago 7591538, Chile; 3 Pedagogy in Physical Education and Health Career, Department of Health Science, Faculty of Medicine, Pontificia Universidad Católica de Chile, Santiago de Chile 7820436, Chile; 4 Hospital Mauricio Heyermann, Servicio de Psiquiatría, Angol 4650207, Chile; 5 Departamento de Educación Física, Deportes y Recreación, Pedagogía en Educación Física, Facultad de Educación y Ciencias Sociales y Humanidades, Universidad de La Frontera, Temuco 4780000, Chile

**Keywords:** executive function, inhibitory control, working memory, cognitive flexibility, sport, older adults

## Abstract

Background: Moderate-to-vigorous intensity exercise programs have proven to exert positive effects on the cognitive performance of older people. However, the specific effects sport-based exercise programs have on cognitive performance, upon executive functions, remain unclear. Therefore, the purpose of this study is to clarify the effects of sport-based exercise programs on executive functions in older adults, through a systematic review protocol of the scientific literature, with a meta-analysis. Methods: The search was performed in the Web of Science, PubMed, Scopus, and EBSCO electronic databases by combining keywords and different medical subject headings (MeSH) to identify and evaluate the relevant studies from inception up until June 2022. This study considers longitudinal studies with at least one experimental group and pre- and post-intervention measurements involving healthy older adults of 60 years of age or older. Studies have to consider one or more measures of executive function, including dimensions of working memory, inhibition, and cognitive flexibility, in order to meet the eligibility criteria for inclusion in this report. The Physiotherapy Evidence Database (PEDro) scale was used for methodological quality assessment studies. The DerSimonian and Laird random-effects model was used to compute the meta-analyses and report effect sizes (ES, i.e., Hedges’ g) with 95% confidence intervals (95% CIs), and a statistical significance set at *p* ≤ 0.05. The ES values were calculated for executive function globally and for each dimension of executive function (e.g., working memory, inhibition, cognitive flexibility) in the experimental and control/comparator groups using the mean and standard deviation values before and after the intervention period. Conclusions: Our systematic review aims to clarify the effects of sport-based exercise programs on executive functions in older adults. The results may help practitioners and stakeholders to provide better evidence-based decisions regarding sport-based exercise program implementation for older adults, and to help them to optimize cognitive functions during the aging process. Ethical permission is not required for this study. Systematic review registration: this systematic review is registered with the International Prospective Register of Systematic Reviews (PROSPERO; registration number: CRD42022284788).

## 1. Background

Executive functions are a set of mental processes that allow thoughts and actions to be regulated during goal-directed behavior, and are responsible for the monitoring and control of those mechanisms that mediate the use of information [1]. Consensus exists on their classification into three central dimensions as follows: inhibition, working memory, and cognitive flexibility [2,3]. On this basis, other higher-order executive functions are organized, such as reasoning, problem solving, and planning [4]. Inhibition allows us to control attention, behavior, thoughts, and/or emotions in order to override a strong internal predisposition to follow impulses, give conditioned responses, or act under the control of environmental stimuli, overcoming distraction or interference from irrelevant information arising from the environment or memory [1,5]. Working memory enables the retention of information in the mind and helps perform cognitive operations using the retained information, thereby manipulating verbal or non-verbal (visual–spatial) content [6]. Cognitive flexibility refers to the ability to appropriately and efficiently adapt our behavior in response to changes in the environment [7]. These skills are important in many aspects of an older adult’s life, including mental and physical health, social development, and maintaining functional autonomy [8,9,10].

Executive functions are associated from an early age with physical activity [11,12,13,14,15] and, particularly, with the practice of sports [16,17,18,19,20]. Such a relationship has also been evidenced in older people, and associations have been reported between complex motor tasks and executive functioning [21]. In fact, some research suggests that older adults use a higher-order set of cognitive skills, such as executive functions, to help execute complex motor tasks, given that motor control may become less automated with age [22,23,24]. However, it is necessary to consider that both cognitive and motor skills undergo changes during this stage of life [25,26], which necessitates the implementation of strategies that enable an individual to cope with normal functional decline [27,28].

Physical exercise is described as an intervention that can provide cognitive benefits for maintaining brain health among older adults [29,30,31,32,33]. The evidence points out that chronic (extending over weeks, months, or years) moderate-to-vigorous intensity (50–75% heart rate reserve) [34] exercise programs elicit favorable effects on cognitive performance in cognitively-normal older adults [35]. This can vary based on aspects such as sex and on the cognitive modality assessed, with larger effects being observed in those measures associated with the executive function and in studies with a higher percentage of women [35,36,37,38]. The moderating effect of sex, however, has not been evidenced in other studies investigating the effects of physical training on executive function and possible underlying moderators in cognitively normal older adults and those with mild cognitive impairment [39,40]. The mechanisms that have been described to mediate such an effect include the influence that prolonged exercise has on the expression of neurotransmitters, neurotrophic factors, synaptic plasticity, the modification of inflammatory pathways, and cerebrovascular function [35,41,42]. 

The types of exercise typically employed in chronic intervention programs among the elderly include aerobics, resistance training, mind–body exercise, and multimodal exercise (combining aerobic exercise and resistance training, among others), which have been followed up by systematic reviews and meta-analyses that investigated the specific effects of these modalities on executive functions [40,43,44]. In this regard, Zhidong et al. [43] proved that the type of exercise can act as a moderator on the effect found on executive functions among older people. Xiong et al. [44] reported that >13 weeks of aerobic exercise significantly improves working memory performance and cognitive flexibility in cognitively healthy older adults, whereas interventions of >26 weeks, significantly improved inhibition. Training programs using mind–body exercises have revealed the presence of significant positive effects on working memory performance and cognitive flexibility, which have been observed to increase when there is a higher frequency of group practice and additional practice at home (more or equal to five times a week) [40,44]. However, some limitations from previous reviews and meta-analyses have been proclaimed, which should be considered in future research, such as the search limitation (in terms of the year of publication of the studies included, or the language), and the consideration of a limited number of measures for the main dimensions of the executive functions (working memory, inhibition, and cognitive flexibility), which can generate a potential bias in the results obtained [39,43].

In terms of its benefits on executive function in older people, a type of intervention that has not been specifically assessed in previous reviews thus far are those which are structured upon a sport modality, such as combat sports (taekwondo or karate) [45,46,47,48,49], individual sports (swimming, table tennis, golf, or Nordic walking) [50,51,52,53,54], or adapted team sports, such as walking football [55]. Pacheco et al. [45] studied the effectiveness of Karate-Do training on cognition among healthy older adults, revealing significant improvements in visual memory and cognitive flexibility after a 12 week intervention period. However, Cho and Roh [46], assessed the effects of regular taekwondo training for 16 weeks on physical fitness, neurotrophic growth factors, cerebral blood flow velocity, and cognitive function among healthy elderly women, revealing the presence of a significant increase in inhibitory control performance after the period of intervention, which could be attributable to increased levels of neurotrophic growth factor. 

While physical activity involves movements producing an increase in energy expenditure (due to increased skeletal muscle activity) above resting conditions, exercise (physical exercise) usually entails specific movement patterns performed systematically over a planned schedule to achieve a desired aim in line with improvement of fitness and health-related outcomes [56]. Regarding sport-based activities, these involve movements with defined goals, containing explicit formal rules, and structured relationships between participants–athletes [57]. Sport is particularly fundamental as it meets a series of conditions that allow for the enhancement of the executive functions of those who practice it, incorporating complex, controlled, and varied movements, which in turn demand various degrees of adaptation to the environmental requirements [58,59,60,61]. In addition, learning in sports is attractive and favors the progressive achievement of aims, thereby promoting emotional and social development, especially when its practice involves contact with other people or being part of a team [62,63,64,65]. The aforementioned aspects could bring sport closer to simultaneous exercise–cognitive training, considering that studies analyzing the effects of aerobic training combined with cognitive challenges are associated with cognitive improvements in healthy older adults, especially regarding executive function, which can prolong functional autonomy among older adults [66,67,68].

Therefore, we present this protocol to collect, in a systematic manner, as much scientific evidence as possible, and to analyze the effects of sport-based interventions on the main dimensions of executive functions in healthy older adults.

## 2. Materials and Analysis

### 2.1. Review Question

What are the effects of sport-based interventions, compared with an active or passive control condition, on the main dimensions of the executive functions in healthy older adults?

### 2.2. Search Strategy

This protocol was prepared pursuant to the guidelines established by Preferred Reporting Items for Systematic reviews and Meta-Analysis Protocols (PRISMA-P) [69]. 

The following electronic databases were used: Web of Science, PubMed, Scopus, and EBSCO through a combination of different medical subject headings (MeSH) or synonyms aimed at identifying and assessing relevant studies from inception to June 2022, using no filters or limits to conduct the search (for example, regarding language or publication date). To visualize the specific search strategy in each database, see Appendix A.

Additionally, reference lists of previous reviews and all trials included were manually searched to identify potentially eligible studies. Systematic reviews were searched for in the same databases with the filters (or terms) “systematic review” OR “reviews” after the regular search strategy. We consulted two external experts in executive functions (who have a PhD and publications in indexed journals) to check the inclusion list of articles and to identify possible articles that could be missing in the list. The experts were included based on Expertscape rank for “Executive + function” which can be found in the link: https://www.expertscape.com/ex/executive+function (accessed on 22 July 2022).

### 2.3. Eligibility Criteria 

The eligibility criteria were defined based on PICOS, a summary of which is presented in Table 1.

Studies published as original articles in peer-review journals were selected. The studies had to be available in full text and contain enough data to calculate effect sizes (ES).

Eligible studies were required to report pre–post intervention change for one or more measures associated with the executive functions of working memory, inhibition, or cognitive flexibility. These tasks must be directly applied to the participants through validated instruments for the respective population. Some examples of tasks that the studies include are the N-back task [70] to evaluate working memory, Stroop task [71] to assess inhibition, and the Trail Making Test—Part B [72] to measure cognitive flexibility.

Eligible studies include chronic intervention programs based on a sport with a minimum duration of 4 weeks. According to previous studies [45,46,47,49,50,51], 4 weeks is probably the minimal effective dose (for duration) for executive function adaptations in older adults after a physical exercise program. 

### 2.4. Data Management

Articles were imported into a reference management system where duplicates were removed.

Two independent authors (FCO and CCJ) examined the titles and abstracts of the articles found in the databases, applying the inclusion criteria using yes and no instructions. If discrepancies arose between the authors, the assessment was discussed with a third author (RRC), who collaborated with them to reach a consensus.

The bibliography of other previous reviews and of the studies finally selected were reviewed following the same process described above to identify possible new studies that met the inclusion criteria. A PRISMA flowchart was used [69] to document the selection process, in addition to the reasons for exclusion, when applicable.

### 2.5. Data Extraction

One reviewer (FCO) independently completed the data extraction, which was verified by a second reviewer (CCJ), regarding the following aspects: author, year of publication, sample size, characteristics of the participants (sex, age, years of schooling and sports experience), description of the sports training program (structure or stages), sport, intervention length, weekly frequency, session length and intensity, control condition, dimensions of the executive function assessed, tasks used, reported reliability indices, and description of the measurement protocol used in each study.

The means and standard deviation of the dependent variables just before and after the sport-based interventions of the studies included were extracted using Microsoft Excel (Microsoft Corporation, Redmond, WA, USA). If the required data were not communicated in a clear or complete manner, the authors of the respective study were contacted for clarification. If no response was obtained from the authors (after two attempts within a 2 week period) or they were unable to provide the requested data, the study result was excluded from the analysis. In cases where data were presented in a figure and the authors did not provide numerical data after being contacted, a validated software was used (r = 0.99, *p* < 0.001) [73] (WebPlotDigitizer, version 4.5, Pacifica, CA, USA; https://apps.automeris.io/wpd/) (accessed on 29 July 2022) to derive the numerical data of the figures by two independent authors (FCO and CCJ), after which Cronbach’s Alpha was calculated. Two authors (FCO and CCJ) performed the data extraction independently, and any interauthor discrepancies (e.g., mean value for a given outcome, number of participants in a group) were resolved by consensus with a third author (RRC). 

### 2.6. Risk of Bias (Quality) Assessment

The Physiotherapy Evidence Database (PEDro) scale was used to assess the methodological quality of the studies included, which was rated from 0 (lowest quality) to 10 (highest quality). Validity and reliability of the PEDro scale has been determined previously [74,75,76]. In studies, its items mostly assess factors related to the risk of bias. Accordingly, it helps to make comparisons between meta-analyses. The methodological quality of studies was interpreted using the following convention [77,78,79]: ≤3 points were considered “poor” quality, 4–5 points were considered as “moderate” quality, and 6–10 points were considered “high” quality. For trials which were already rated and listed in the PEDro database, the respective scores were adopted. Two authors (FCO and CCJ) assessed the methodological quality for each study included independently, and any discrepancies between them were resolved via consensus with a third author (RRC).

All studies meeting the inclusion criteria were included in the review, regardless of their methodological quality. However, this aspect was considered in the interpretation and discussion of the results. 

### 2.7. Strategy for Data Synthesis

Although meta-analyses can be performed with as few as two studies [80], because reduced sample sizes are common in sport-science literature [81], meta-analysis was only conducted when >3 studies were available [82,83]. Effect sizes (ES, i.e., Hedges’ g) were calculated for executive function globally and using the mean and standard deviation before and after the intervention period for each dimension of executive function in the experimental and control/comparator groups. If studies reported data other than mean and/or standard deviation values, appropriate statistical conversion was performed before the meta-analysis. Data was standardized using post-intervention standard deviation values. The DerSimonian and Laird random-effects model was used to account for those differences identified between the studies that might affect the intervention effect [84]. The ES values were presented with 95% confidence intervals (95% CIs). Calculated ES was interpreted using the following scale: <0.2 trivial, 0.2–0.6 small, >0.6–1.2 moderate, >1.2–2.0 large, >2.0–4.0 very large, >4.0 extremely large [85]. In studies involving more than one intervention group, the sample size in the control group was divided proportionally to facilitate comparisons between the multiple groups [86]. The level of heterogeneity was assessed using the I² statistic, with values of <25%, 25–75%, and >75% representing low, moderate, and high levels of heterogeneity, respectively [86]. The risk of publication bias was explored for continuous variables (≥10 studies per outcome) [87,88] using the extended Egger’s test [87]. To adjust for publication bias, a sensitivity analysis was conducted using the trim and fill method [89], with L0 as the default estimator for the number of missing studies [90]. All analyses were performed using the Comprehensive Meta-Analysis software (version 2, Biostat, Englewood, NJ, USA). Statistical significance was set at *p* ≤ 0.05. 

## 3. Discussion

Previous systematic reviews focused on aerobic-based or coordination-based exercise interventions and their effect in older adults’ executive function [39,43,44]. However, given the potential of sport-based exercise interventions in older adults’ executive function and the considerable literature currently available on the topic, our systematic review aims to clarify the effects of sport-based exercise programs on the executive function of older adults. Results may help practitioners and stakeholders to provide better evidence-based decisions regarding sport-based exercise programs implementation in older adults, and to help them to optimize cognitive functions during the aging process. 

Participation in sports activities is an opportunity to improve physical condition and enhance executive functions in an attractive way for older people as long as the activities respond to their interests and special importance is given to the safety of the participants (for example, considering a previous medical evaluation), avoiding possible sports injuries, or adverse effects. 

It is necessary to consider that learning new skills is an excellent tool for developing executive functions, as the person must attend to and manipulate new information, responding efficiently to the requirements of the environment, while favoring social relations. It is undoubtedly a challenge to motivate older people to break their routines and start new activities, but for many of them it could be an opportunity to resume a sport practiced at another stage of life and an instance that allows them to commit to new challenges. 

Final conclusions can be drawn with regards to the effects of sport-based programs on each of the main executive functions analyzed: working memory, inhibition, and cognitive flexibility. Limitations that emerge from this study are discussed in detail and possible lines of research on this subject have been considered.

The methodological analysis of the studies included contribute to research in this field due to ascertaining aspects that require improvements in future studies. Finally, the results obtained are disseminated to various audiences with the aim of benefiting older people with this information and thus contributing to their improved quality of life. 

## Figures and Tables

**Table 1:** Eligibility Criteria Based on PICOS.

PICOS	Inclusion Criteria	Exclusion Criteria
Population	Healthy older adults (mean age, ≥60 years) without restrictions based on sex or fitness level.	Children, adolescents, or middle-aged adults. Individuals with a medical condition that may limit their participation in sport-based activities, meaning that they must not have any neurological pathology, psychiatric disorder, or other types of medical conditions. Participants of paralympic sports or individuals with disabilities are not included.
Intervention	Chronic intervention programs (with a minimum duration of 4 weeks) based on a sport, of a competitive or recreational type. The interventions should involve sport exercises (e.g., soccer) or sport-based or sport-adapted exercises (e.g., walking soccer).	Acute interventions. Chronic sport-based interventions combined with different types of exercises (for example, aerobics or resistance training) or with the aid of a nutritional supplement. Chronic interventions that are not related to a sport.
Comparator	Group not exposed to the sports training program. The control group may be active (alternative training method, such as balance or stretching program) or passive (continuing their usual activities of daily living).	Absence of control group.
Outcome	Pre-post-intervention values for one or more direct assessment measures for the executive functions of working memory, inhibition, or cognitive flexibility.	Indirect measures of executive functions (e.g., questionnaire). Measures of executive functions other than working memory, inhibition, or cognitive flexibility.
Study design	Longitudinal studies with at least one experimental group and a control group, that include pre- and post-intervention measurements.	Cross-sectional studies; Single-group interventions.

## Data Availability

Not applicable.

## Appendix A

**Table A1:** Specific Search Strategy for Each Database.

Database	Specificities of the Database	Search Strategy
EBSCO	EBSCO does not allow combinations of title and abstract. To avoid multiple internal combinations, we decided to use a more open search strategy in this database, with all code lines being open to “All text”.	TX (“executive functions” OR “cognitive functions” OR cognition OR “inhibitory control” OR inhibition OR “working memory” OR shifting OR “cognitive flexibility”) AND TX (sports OR “modified sport” OR exercise OR “physical activity” OR athletics OR “sport practice”) AND TX (“older adults” OR aging OR elderly OR aged OR “older people”)
PubMed	Nothing to report.	((“executive functions” [Title/Abstract] OR “cognitive functions” [Title/Abstract] OR cognition [Title/Abstract] OR “inhibitory control” [Title/Abstract] OR inhibition [Title/Abstract] OR “working memory” [Title/Abstract] OR shifting [Title/Abstract] OR “cognitive flexibility” [Title/Abstract]) AND (sports [Title/Abstract] OR “modified sport” [Title/Abstract] OR exercise [Title/Abstract] OR “physical activity” [Title/Abstract] OR athletics [Title/Abstract] OR “sport practice” [Title/Abstract])) AND (“older adults” [Title/Abstract] OR aging [Title/Abstract] OR elderly [Title/Abstract] OR aged [Title/Abstract] OR “older people” [Title/Abstract])
Scopus	In Scopus, the search for title or abstract also includes keywords.	TITLE-ABS-KEY (“executive functions” OR “cognitive functions” OR cognition OR “inhibitory control” OR inhibition OR “working memory” OR shifting OR “cognitive flexibility”) AND TITLE-ABS-KEY (sports OR “modified sport” OR exercise OR “physical activity” OR athletics OR “sport practice”) AND TITLE-ABS-KEY (“older adults” OR aging OR elderly OR aged OR “older people”)
Web of Science	In Web of Science, the search for title or abstract also includes keywords, and is termed “topic”.	((TS = (“executive functions” OR “cognitive functions” OR cognition OR “inhibitory control” OR inhibition OR “working memory” OR shifting OR “cognitive flexibility”)) AND TS = (sports OR “modified sport” OR exercise OR “physical activity” OR athletics OR “sport practice”)) AND TS = (“older adults” OR aging OR elderly OR aged OR “older people”) https://www.webofscience.com/wos/woscc/summary/1e82b09c-85e7-4c70-92d9-60af5b107fd0-48df71c9/relevance/1 (accessed on 1 July 2022)
